# Identification and Characterization of Three *Epichloë* Endophytes Isolated from Wild Barley in China

**DOI:** 10.3390/jof11020142

**Published:** 2025-02-13

**Authors:** Zhengfeng Wang, Feng Zhao, Qijun Bao, Xiaoning Liu, Cheng Guo

**Affiliations:** 1 Economic Crop and Malt Barley Research Institute, Gansu Academy of Agricultural Science, Lanzhou 730070, China; 2 Plant Protection Research Institute, Gansu Academy of Agricultural Science, Lanzhou 730070, China

**Keywords:** wild barley, *Epichloë bromicola*, morphology, phylogenetic analysis, alkaloid biosynthetic genes

## Abstract

*Epichloë* endophytes have been found in cool-season grasses and can produce alkaloids that are toxic to vertebrates and insects. Due to their beneficial effects, *Epichloë* can provide plants with resistance to some abiotic and biotic stresses. The biological and physiological characteristics of the endophytic strains XJE1, XJE2, and XJE3 isolated from wild barley were measured across a range of pH, salt concentrations, and growth values. The phylogenetic position of the *Epichloë* isolates was examined using the *tefA* and *actG* genes. The optimal pH values for mycelial growth of XJE1, XJE2, and XJE3 were 7–8, 6–7, and 8–9, respectively. The isolates grew significantly better at 0.3 mol/L NaCl than at 0.5 mol/L and 0.1 mol/L NaCl. Based on the conidiophore and conidia morphology, growth characteristics, and phylogenetic relationships, the endophyte isolated from wild barley is likely *Epichloë bromicola.* These isolates exhibited differences in mating types and alkaloid biosynthesis genes. Screening for salt tolerance and alkaloid biosynthetic genes in endophytic strains will provide new insights into useful traits to breed into new forage germplasms.

## 1. Introduction

Endophytic fungi are common and diverse in grasses [1,2]. They are mainly distributed inside the leaf sheath and on the seeds, flowers, stems, leaves, and roots of grasses. They often colonize above-ground tissues of the grass plant [3]. The relationship between endophytic fungi and their hosts can be mutually beneficial, or it can be parasitic, neutral, or temporarily harmless to the host [4]. *Epichloë* fungi consist of a small number of clavicipitaceous species that only exist in grasses grown in temperate regions [5]. The fungi colonize the host plant year-round in the form of mycelia. They do not produce spores, but grow mycelia into the reproductive organs of the host plant and then spread with the seeds [6]. Most *Epichloë* endophytes can produce a large number of secondary metabolites inside the infected host, such as indole acetic acid, ergovaline, and polylamine, which can increase the number of tillers, promote growth, and improve the resistance of the host grass to pests and herbivores [7,8,9]. There are many reports on the species, genetic, and host diversity of *Epichloë* fungi and the mechanisms that enable *Epichloë* fungi to enhance host stress resistance [10,11,12]. *Epichloë* fungi are very diverse, and taxonomic methods enable scientists to understand the diversity of these organisms [13]. In fungal classification, morphological, physiology, and molecular characteristics are usually examined.

In recent years, molecular techniques have played an increasingly important role in the classification of *Epichloë* fungi species, but traditional morphological observation methods are still important in taxonomic identification work [14,15]. The morphological, physiological, and phylogenetic characteristics of *Epichloë* fungi in grasses have been reported for *Epichloë coenophiala* [16], *Epichloë bromicola* [17], *Epichloë gansuensis* [18], and *Epichloë sinensis* [19]. The mycelial biomass and the types of nitrogen and carbon sources consumed by *Neotyphodium coenophialum* [20], isolated from tall fescue, were examined by growing five *Neotyphodium* species on media containing five sugars. Yuping Zhang [21] studied the biological and physiological characteristics of *Neotyphodium* endophytic fungi isolated from *Elymus dahuricus* Turcz. Li et al. [18] reported on the biological and physiological characteristics of the new species *Neotyphodium gansuense*.

Wild barley (*Hordeum brevisubulatum*) is an excellent perennial forage grass that is distributed in Eastern Europe, Central Asia, Siberia, Iran, Pakistan, China, and other places. In China, it is abundant in the Northeast region, Xinjiang, Inner Mongolia, Gansu, Ningxia, Northern Shanxi, Qinghai, and Tibet. Wild barley is rich in nutrients, soft, and palatable and has the ability to grow in drought and high-salt environments. The endophytic *Epichloë* fungus was first discovered in wild barley in 1991 [22]. The infection rate of wild barley in Xinjiang and Gansu with the fungus is 100%, and its infection rate in Inner Mongolia is 67–90% [23]. In addition, greenhouse experiments showed that endophytic fungal infections significantly increase plant growth, with the biomass, above-ground matter, and root dry weight increasing by 36.4%, 33.3%, and 30.0%, respectively. Numerous studies have found that endophytic fungi improve insect resistance, drought tolerance, and salinity tolerance in wild barley [24].

A large number of studies have suggested that the biologically active alkaloids produced by certain species of *Epichloë* endophytes can improve the plant’s defenses and can deter chewing insects [8]. *Epichloë* species have been shown to produce four classes of alkaloids, based on genetic analysis: peramine, lolines, ergots, and indole diterpenes. Other studies have shown that peramine can affect insects’ nerve receptors, resulting in neurotoxic effects that prevent insects from feeding [5]. Conversely, certain insects have evolved the ability to avoid, resist, and even exclude peramine to bypass its toxic effects. Interfering with insect feeding by applying peramine may significantly increase the competitiveness of fungus-infected grasses [3]. In addition, other advantages of expressing peramine in the host grasses included chemosensitization and suppression of plant pathogens and seed feeders [5]. In that study, all genes involved in the biosynthesis of peramine were detected using multiplex PCR.

This study aims to provide a basic understanding of endophytic fungi in wild barley collected in Tachen, Xinjian province. The results obtained from the study will lay the foundation for further research on wild barley–endophyte symbiosis. Thus, in this study, a detailed characterization of *Epichloë* fungal isolates from wild barley was conducted using morphology and molecular methods, and their bioactive potential was explored.

## 2. Materials and Methods

### 2.1. Plant Collection and Endophyte Isolation

*Hordeum brevisubulatum* plants were collected from the Xinjiang region (N 87°57′56″, E 43°67′83″, China). Three sampling sites were selected with consistent growth of wild barley, and 10 well-developed whole plants were taken from each sampling site. The fungal hyphae inside all the collected plants were stained with aniline blue, and the endophyte infection rate was assessed under a microscope (Tokyo, Japan, Olympus, DM-125) [25]. Fungal endophytes were isolated from seeds following the method described by Wang et al. [24]. In brief, the seeds were surface-disinfected by rinsing them in 75% ethanol for 3 min, then in 5% sodium hypochlorite for 3 min, and finally washing five times with sterile distilled water. The treated seed samples were dried with sterilized filter paper and placed on PDA (potato dextrose agar medium, Tianjing, China) plates that were then sealed with parafilm and incubated at 22 ± 2 °C for 2–3 weeks [26]. When the endophyte colonies appeared on the plates, they were transferred to fresh PDA plates and incubated for 2 weeks before the fungi were examined morphologically.

### 2.2. Morphological Identification

To examine the hyphal structures of the endophytes, fungal colonies 0.5 cm in diameter were transferred to PDA and incubated in the dark at 22 °C for 4 weeks. A sterilized core borer (aperture 6 mm) was used to punch a hole into the fresh mycelium on the medium. The fungal mycelia were transferred onto PDA agar plates. The pH of the PDA was adjusted to 4, 5, 6, 7, 8, 9, or 10 with 1 mol/L NaOH and HCl. The pH value of the culture filtrate was determined with a pH meter (PB-20 type). The NaCl concentration of the PDA was adjusted to 0.1 mol/L, 0.3 mol/L, or 0.5 mol/L. The petri dishes containing solid culture medium (9 cm) were each coated with one fungal mycelium (in triplicates) and cultured at 22 ± 2 °C for 4 weeks. The fungal colony diameter was measured by the ‘cross’ method. Morphological characteristics, such as the colony’s color, diameter, and growth rate, were examined. The conidia and conidiogenous cells were measured and photographed under a microscope after inoculating 1.5% water agar plates with the fungi, as described in [24]. The length of the conidiogenous cells (*n* = 50) and the width and length of the conidia (*n* = 50) were measured using an automated upright fluorescence microscope (Olympus Corporation, Tokyo, Japan, Olympus, BX63).

### 2.3. Molecular Identification

After 2 weeks of growth on PDA at 22 °C, the fungal mycelia were collected in Eppendorf tubes. The fungal DNA was extracted using an HP fungal DNA kit (OMEGA, Beijing, China) as described in the manufacturer’s instructions. Two different primer pairs were selected for PCR amplification of the following phylogenetic markers: *tefA* (tef1-exon 1d-1, 5′-GGGTAAGGACGAAAAGACTCA, and act1-exon 6u-1, 5′-AACCACCGATCCAGACAGAGT) and *actG* (act1-exon 6u-1, 5′-AACCACCGATCCAGACAGAGT, and act1-exon 5u-1, 5′-TAATCAGTCACATGGAGGGT) [26]. The PCR conditions were set as follows: an initial denaturation step of 5 min at 94 °C followed by 30 cycles of 30 s at 94 °C, annealing for 50 s at 55 °C (*tefA*) and 45 °C (*tubB*), and a final elongation step of 10 min at 72 °C. The PCR products were checked via electrophoresis on 2% agarose gels. DNA sequencing was performed by Bioengineering Co. (Shanghai, China). Sequence analysis of the endophytic fungi was conducted using the National Center for Biotechnology Information (NCBI) database. Multiple sequence alignment was performed using CLUSTALW (https://www.genome.jp/tools-bin/clustalw, accessed on 4 December 2024). MEGA-11 was used to select the optimal model and calculate the maximum likelihood phylogenetic trees with 1000 bootstrap replications. The unique sequence data from this study were submitted to GenBank, and the accession numbers are *tefA* (OR397472, OR397473, and OR397474) and *tubB* (OR397477, OR397478, and OR397479).

### 2.4. Alkaloid Gene Identification

PCR was performed to test fir the presence of genes associated with the synthesis of peramine, indole-diterpenes, ergot, and loline alkaloids in the isolated strains XJE1, XJE2, and XJE3, using primers specific to each alkaloid biosynthetic gene, as described previously by [27,28]. The PCR cycling parameters were described by [24,26] *Epichloë* sp. FS001 and *E. inebrians* E818 were used as the positive controls for genes related to lolitrem B and the EAS locus [29]. *E. festucae* var. lolii AR1 was used as the positive control for the perA gene and some of the genes at the IDT locus [30,31].

### 2.5. Statistical Analysis

In addition to the molecular and alkaloid analysis methods and the software already described, IBM SPSS Statistics version 19.0 (SPSS, Chicago, IL, USA)was used to calculate significant differences among the treatments (Duncan method). Differences were considered to be significant at *p* < 0.05.

## 3. Results

### 3.1. Morphological Characteristics of Endophytes

**Figure 1:**
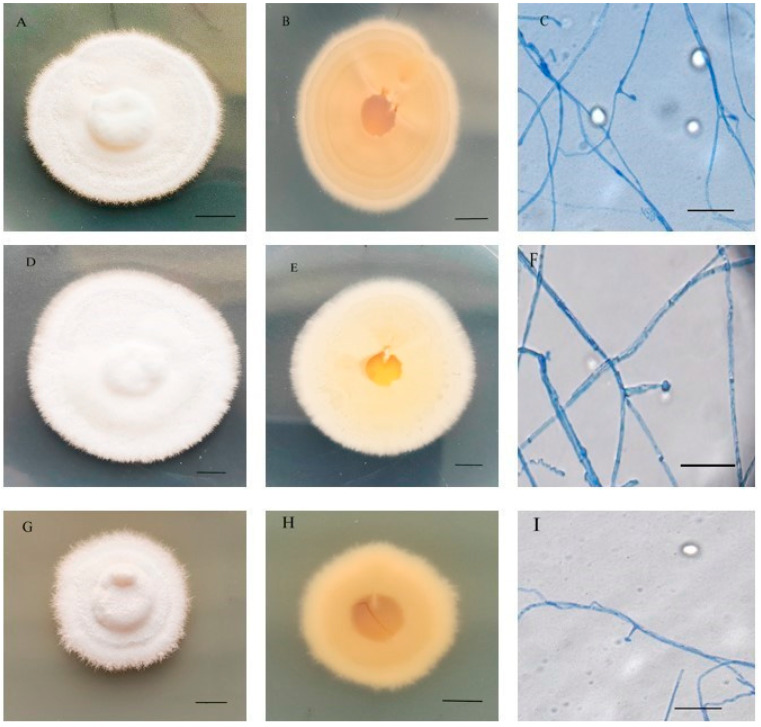
Colony characteristics of XJE1, XJE2, and XJE3 endophyte fungus isolates from wild barley. Obverse of the colony (**A**,**D**,**G**), Bar = 1cm; reverse of the colony (**B**,**E**,**H**), Bar = 1cm; hyphae, conidiophores, and conidia (**C**,**F**,**I**) Bar = 20μm.

After 4 weeks of incubation at 25 °C on PDA medium, the colony diameters of XJE1, XJE2, and XJE3 were 28–42 mm, 22.4–30.8 mm, and 16.8–25.2 mm, respectively. The colony surfaces had a white, fluffy, and compact texture, with a central uplift or slight wrinkling, and the underside was white to yellow (Figure 1A,B,D,E,G,H). The mycelia of XJE1, XJE2, and XJE3 were elongated, branched, and separated (Figure 1C,F,I). The lengths of the conidiogenous cells were 7.6–8.9 μm, the conidia were navicular or reniform, and the conidia size was 3.4–4.5 μm × 3.3–4.8 μm (Table 1).

A comparison of the morphological characteristics of the isolates with reported strains belonging to the *Epichloë* genus showed that the fungal isolates from *H. brevisubulatum* were typical of those within the *Epichloë* genus, but that the strains were morphologically somewhat different from the previously reported strains. The isolated strains could be distinguished from the 13 reported *Epichloë* fungi by the following characteristics (Table 1): the conidiophores and conidia of the strains were similar to the three species *Epichloë* sp. HboTG-2, *Epichloë typhina,* and *Epichloë scottii* and significantly different from the other species. In addition, the shapes of the conidia of the different *Epichloë* strains vary from oval, navicular, and reniform to semilunar, and those of the strains isolated in this study were oval and reniform. The endophytic fungal isolates from wild barely had colony growth rates and morphologies that were typical of *Epichloë* species.

### 3.2. Physiological Characteristics of Fungal Endophytes

At pH 8, the mycelia of XJE1 and XJE3 grew faster than at other pH values, with a colony diameter of 4.2–5.0 cm after 4 weeks of incubation (*p* < 0.05). The isolate of the XJE2 strain grew significantly (*p* < 0.05) better at pH 7 than at pH 4–6 and pH 8–10. The colony diameters of XJE3 grown at pH 8–9 were significantly (*p* < 0.05) larger than those grown at other pH values. The colony diameters of all three isolates grown on alkaline medium were significantly greater those grown on acidic medium (*p* < 0.05). Taken together, the endophytic fungal strains XJE1, XJE2, and XJE3 of wild barley were adapted to grow at a wide range of pH values, and grew optimally at pH 7–8 (Table 2). The results indicate that the three *Epichloë* strains were able to grow at a wide pH range from slightly alkaline to acid conditions (Table 2).

The *Epichloë* strains XJE1, XJE2, and XJE3 were able to grow at NaCl concentrations of 0.1, 0.3, and 0.5 mol/L. The colony diameters at an NaCl concentration of 0.3 mol/L were significantly greater those at 0.1 and 0.5 mol/L (*p* < 0.05). The *Epichloë* strain XJE3 displayed better salt tolerance than the strains XJE1 and XJE2 (Table 3). Therefore, the three *Epichloë* strains were found to be salt-tolerant, and the XJE3 strain was more salt-tolerant than strains XJE1 and XJE2 (Table 3).

### 3.3. Phylogenetic Analyses

**Figure 2:**
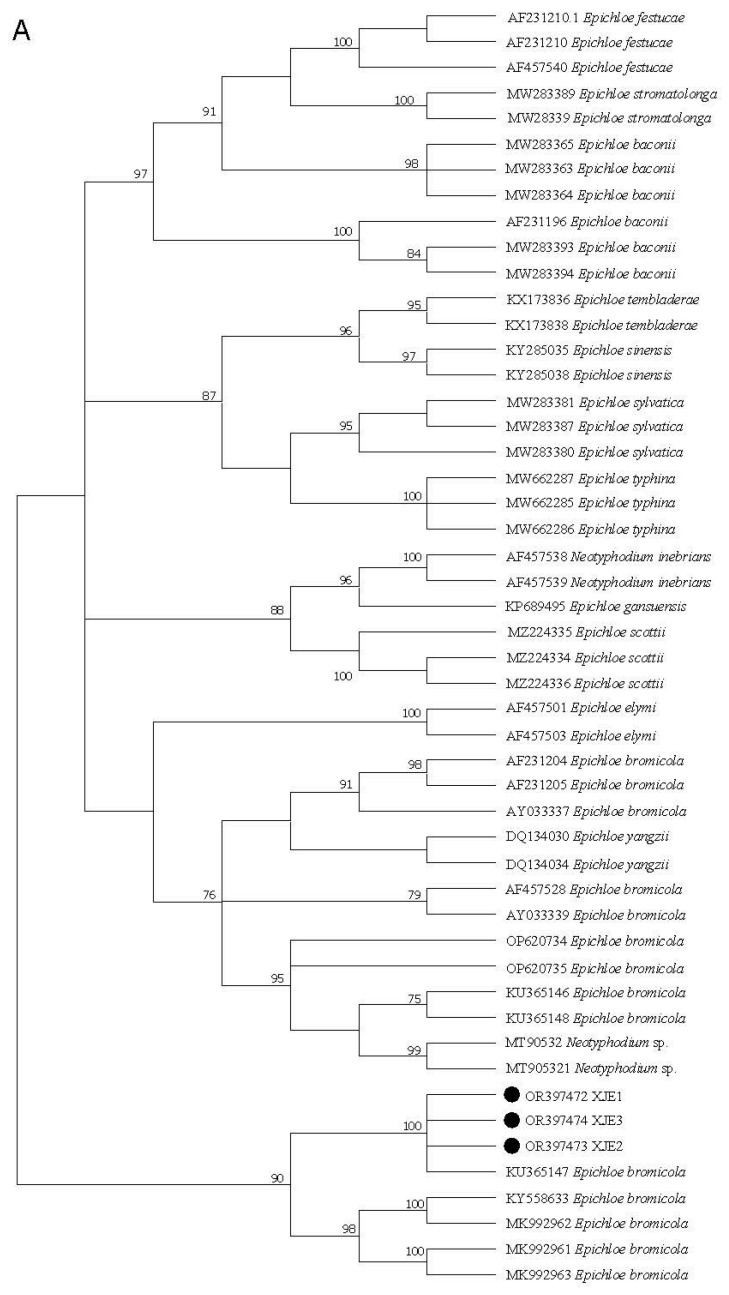

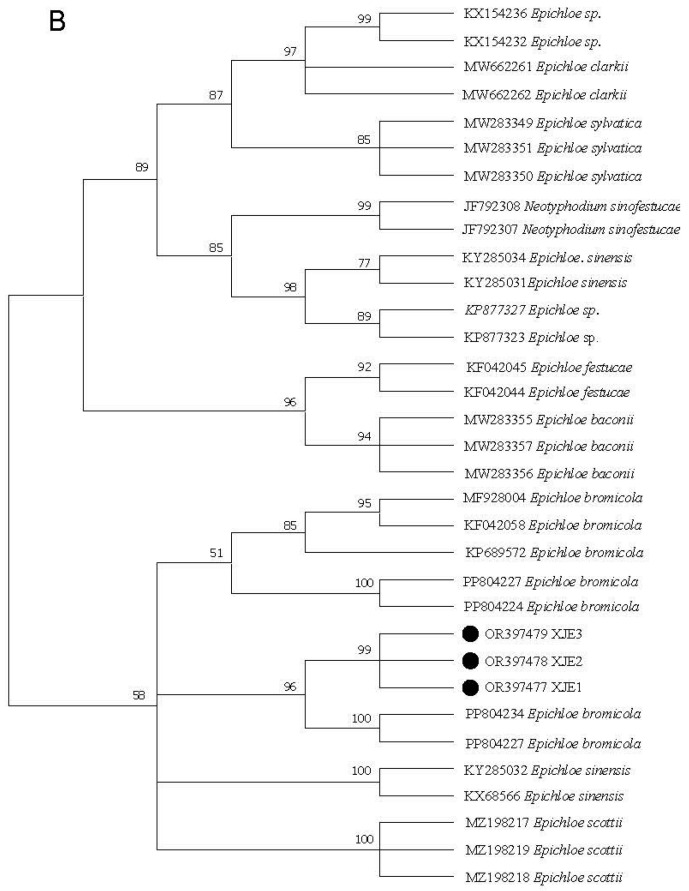
Maximum likelihood (ML) tree of the *tefA* (**A**) and *tubB* (**B**) regions from representative *Epichloë* species and the endophytic strains XJE1, XJE2, and XJE3 isolated from *H. brevisubulatum*. Rooted 50% majority rule consensus maximum parsimony phylogenetic tree of the *tefA* (**A**) and *tubB* (**B**) sequences. The node values indicate the bootstrap percentages based on 1000 replications. Sequences are labeled as GenBank accession numbers and *Epichloë* species names. The endophytic strains XJE1, XJE2, and XJE3 isolated from *H. brevisubulatum* are identified by circles next to the GenBank accession numbers.

PCR amplification of *tefA* and *tubB* from strains XJE1, XJE2, and XJE3 yielded products of about 731 bp, 718 bp, and 735 bp and 816 bp, 827 bp, and 838 bp, respectively. BLAST comparison of the sequencing results with the NCBI/GenBank database showed that the isolates were similar to *Epichloë bromicola*. In the phylogenetic tree of the *tefA* region, XJE1 (OR131295), XJE2 (OR397473). and XJE3 (397474) clustered with *Epichloë bromicola* (KU365147) within a single branch. The similarity rate of 100% among the sequences indicated that the fungi were likely closely related to each other (Figure 2A). Furthermore, the phylogenetic trees that were generated using *tubB* showed that the isolates in this study grouped with *E. bromicola*, with bootstrap values of 96%, thereby providing additional evidence that the isolate was closely related to *E. bromicola* (Figure 2B). From the above results, we concluded that the phylogenetic analyses based on the *tefA* and *tubB* sequences showed consistent results, and strains XJE1, XJE2, and XJE3 clustered with other *Epichloë* species. These results also indicated that the isolates from this study belong to *E. bromicola*.

### 3.4. Alkaloid Gene Profiling and Mating Types

The endophytic strains XJE1, XJE2, and XJE3 were tested using PCR for the presence of genes responsible for producing alkaloids. Four major bioprotective alkaloids, including peramine, loline, ergot, and indole-diterpene alkaloid, were detected using different gene-specific primer sets. The results showed that the XJE1 endophyte harbored *mtAC* and *mtBA*, but the XJE2 and XJE3 endophytes only harbored *mtAC*. Therefore, the XJE1 mating type was MTA and MTB, which confirmed it to be a hybrid (Table 4). The endophytic strains XJE2 and strain XJE3 mating types were MTA. When the strain XJE1 was tested for the presence of pomine alkaloid synthesis genes, perA-ΔR* alleles were not detected, but the remaining seven fragments of the *perA* gene (*perA-A1*, *perA-C*, *perA-T1*, *perA-A2*, *perA-M*, *perA-T2,* and perA-R*) were present. Therefore, strain XJE1 is predicted to produce peramine (Table 4). Strains XJE2 and XJE3 contained the *perA-A1*, *perA-C*, *perA-A2,* and *perA-T2* fragments from the perA locus, but *perA-T1*, *perA-M*, perA-R*, and perA-ΔR* were not detected. All three strains contained fragments of the *easF*, *easE*, *easD*, *easA*, *easG*, *cloA*, *lpsB*, *lpsA,* and *easH* genes from the EAS locus, but *dmaW*, *easC*, *easO*, and *easP* were not detected (Table 4), thereby showing that the endophytic fungi are unable to produce ergovaline. Of the 11 genes at the *IDT/LTM* locus that were tested, *idtP*, *idtE,* and *idtJ* were missing from all three strains, thereby suggesting that they cannot activate the lolitrem B, paxilline, and terpendole pathways in host plants (Table 4).

The presence of synthesis genes for loline alkaloids was inconsistent across the three strains. Of the 11 genes at the *LOL* locus, *lolC*, *lolF*, *lolU*, *lolA*, *lolO*, *lolE*, *lolN,* and *lolM* fragments were detected, but the *lolD*, *lolP,* and *lolT* fragments were missing from the strain XJE1. Strain XJE2 only harbored *lolO* at the *LOL* locus, but none of the 11 genes at the *LOL* locus were detected in XJE3. These results indicated that the isolate cannot produce loline alkaloids (Table 4).

## 4. Discussion

Endophytic fungi belonging to the *Epichloë* genus are the most widely studied endophytic fungi at present [38]. *Epichloë* species are endophytes of many cool-season grasses [14]. They have been found in more than 300 species of grasses belonging to 80 genera [36]. Some differences in their morphological characteristics have been observed in different grasses. The species composition and morphological characteristics of the endophytic fungi of the same species of grass have been shown to vary according to the geographic region [39]. For example, 19 strains of endophytic fungi displaying different morphological characteristics were isolated from six different geographically distinct populations of *Festuca ovina* in Central and Eastern Inner Mongolia [13].

An analysis of the morphological diversity of 21 *E. ansuensis* strains from different geographically distinct populations in northwest China and from different tissues of the plants showed that many differences in the colony color, texture, growth rate, conidial size, and conidial shape of these strains existed [40]. Previous studies have also observed that *E. bromicola* strains isolated from *H. brevisubulatum* from two provinces in China displayed minor phenotypic differences [17,24]. In this study, the peduncle lengths of strains XJE1, XJE2, and XJE3 isolated from wild barley were 7.6–8.9 μm, and the conidia were 3.4–4.5 μm × 3.3–4.8 μm. Previous studies have reported the conidial peduncle length and conidia size in *Epichloë* species to be 15–76 μm and 5.0 ± 1.1 μm × 1.7 ± 0.25 μm, respectively. The conidial peduncle length and conidia size range in strains XJE1, XJE2, and XJE3 were similar to those of other *Epichloë* species. *E. bromicola* strains have been found across Eurasia and are reported to have a broad host range, which includes *H. brevisubulatum*, *Elymus* spp., *Bromus* spp., *H. europaeus*, *Hordelymus* spp., *Leymus* spp., *L. chinensis*, and *Elymus* spp. [36,41,42]. The taxonomic identity and geographic distribution of the host plant play important roles in identifying *Epichloë* species. In this study, based on morphological and molecular phylogenetic results, the endophytic fungi isolated from *H. brevisubulatum* were identified as *E. bromicola*.

The isolated strains had good salt tolerance and grew optimally at an NaCl concentration of 0.3 mol/L. The optimal pH value for mycelial growth was pH 7–8. This result was inconsistent with what is reported for *E. gansuense*, for which the mycelia grow faster at pH 5–6 [18]. A study of *Epichloë* spp isolated from *Festuca sinensis* reported the optimal pH value to be pH 5 [29]. Therefore, the same endophytic species may exhibit different biological properties depending on the host species and geographic location. Different species of grass-associated endophytic fungi have also been reported to share similarities in their biological characteristics, such as optimal conditions for mycelial growth among most *Epichloë* spp. on PDA and growth inhibition of endophytic fungi associated with wild barley by xylose, except *E. coenophiala*, *E. chisosa*, and *E. typhina*, which can grow marginally well in the presence of xylose [20,29,40]. This study suggests that *E. bromicola*–infected *H. brevisubulatum* may have in-creased levels of saline–alkali tolerance compared to uninfected plants.

A remarkable feature of *Epichloë* fungi that they can provide direct defense mechanisms to the host by producing protective alkaloids that prevent the host from being foraged by phytophagous insects or herbivores. The three strains were assayed for the *perA* gene clusters, and only strain XJE1 harbored it. Interestingly, Tanaka et al. [43] discovered that peramine was only produced by members of the *Epichloë* genus via a multifunctional non-ribosomal peptide synthetase encoded by the *perA* gene. Peramine biosynthesis was completely disrupted when the functional *perA* was deleted in *E. festucae*. In addition, peramine production is highly influenced by interactions between the endophyte and its host plant [44]. For instance, Charlton et al. [45] detected two alleles of *perA* in *E. cabralii*, but the production of peramine was not detected. Chen et al. [46] also showed that the *LOL*, *EAS,* and *perA* gene clusters were detected in *E. bromicola*, but only peramine accumulation was identified.

Lolines have deterrent effects on plant-feeding insects, such as *Heteronychus arator*, *Rhopalosiphum padi*, *Trigonotylus caelestialium,* and *Pratylenchus scribneri* [3]. Most loline-synthesizing genes are tightly regulated in endophyte fungi, resulting in the accumulation of alkaloids only when they are associated with a host plant [47]. Indole diterpenes and ergot induce neurotoxicity in mammals. For example, grazing on *Epichloë*-infected grass leads to tremorgenic symptoms, such as muscle fatigue, head tremors, and hypersensitivity in mammals [48]. Alkaloid production has been shown to depend on the endophyte associated with the host, and the internal concentration of alkaloids is affected by the plant tissue type, plant physiological state, and climatic conditions [24,46]. In this study, three strains of endophytic fungi showed diversity in alkaloid synthesis genes. However, there are still many unclear mechanisms of alkaloid synthesis and uncharacterized genes. Future research will unravel the mechanisms of alkaloid production by fungi or the factors that affect them and the production of these alkaloids in vitro by endophytes.

## 5. Conclusions

The morphological, physiological, and phylogenetic analyses in this study showed that strains XJE1, XJE2 and XJE3 could be identified as *E. bromicola.* Molecular analysis of those isolates indicated that they were of a hybrid AB mating type. Further studies will yield more insights into the quantitative and qualitative analysis of alkaloids and determine the effects of the strains on plant resistance to biotic and abiotic stress. The optimal pH values for strains XJE1, XJE2, and XJE3 were pH 7–8. The isolates could grow at 0.5 mol/L NaCl, showing that these strains have salt tolerance. Phylogenetic analysis of mating type and alkaloid biosynthesis genes suggested that the XJE1 isolate is an interspecific hybrid species and can produce peramine, which is toxic to insects. To confirm their ability to enhance plant traits, further investigations are needed to identify and measure alkaloids using chromatography and mass spectrometry in in vivo experiments. However, this study provides valuable insights that will facilitate future discoveries of endophytic symbionts.

## Figures and Tables

**Table 1:** Morphological characteristics of *Epichloë* endophyte fungi isolated from *Hordeum brevisubulatum*.

Endophyte	Host	Growth on PDA (mm/day)	Conidia Size (μm)		Length of Conidiogenous Cell (µm)	Origin
			Length	Width		
**XJE1**	*Hordeum brevisubulatum*	1.0–1.5	4.1 ± 0.7	3.4 ± 0.6	8.0 ± 1.5	This study
**XJE2**	*Hordeum brevisubulatum*	1.1–1.3	3.3 ± 0.5	2.8 ± 0.2	8.9 ± 2.0	This study
**XJE3**	*Hordeum brevisubulatum*	1.2–1.4	4.8 ± 0.5	4.5 ± 0.7	7.6 ± 0.9	This study
**XJE4**	*Hordeum brevisubulatum*	1.1–1.4	5.5 ± 0.4	3.8 ± 0.3	9.9 ± 1.2	This study
***Epichloë* sp. HboTG-3 **	*Hordeum bogdanii*	0.9 ± 0.1	7.4 ± 1	4.47 ± 0.4	21.8 ± 5.7	[14]
***Epichloë festucae***	*Festuca rubra*	1.0–2.67	4.7 ± 0.6	2.2 ± 0.3	12.0–25.0	[32]
***Epichloë elym***	*Elymus* sp.	1.9–2.9	2.2 ± 0.2	4.0 ± 0.4	17.0 ± 3.0	[33]
***Epichloë* sp. HboTG-2 **	*Hordeum bogdanii*	0.8 ± 0.1	5.9 ± 1.0	3.1 ± 0.5	17.4 ± 6.2	[14]
***Epichloë typhina***	*Dactylis glomerata*	1.9–3.0	4.1 ± 0.5	2.2 ± 0.5	13.0–33.0	[23]
***Epichloë amarillans***	*Agrostis hiemalis*	nt	4.5–1.9	1.7 ± 0.2	23.4 ± 6.5	[34]
***Epichloë scottii***	*Melica uniflora*	1.0–1.1	4.1 ± 2.8	2.5 ± 0.4	14.1 ± 2.8	[35]
***Epichloë disjuncta***	*Hordelymus europaeus*	0.7–1.3	6.9–2.7	2.0–2.5	22.0–48.0	[36]
***Epichloë hordelymi***	*Hordelymus europaeus*	1.8–2.2	7.6 ± 0.9	2.7 ± 0.3	38.1 ± 9.1	[36]
***Epichloë danica***	*Hordelymus europaeus*	0.5	5.8 ± 0.4	2.4 ± 0.1	23.3 ± 4.1	[29]
***Epichloë sylvatica***	*Hordelymus europaeus*	1.0–1.3	4.6 ± 0.4	2.3 ± 0.2	50.8 ± 6.6	[36]
***Epichloë bromicola***	*Hordelymus europaeus*	1.4–1.6	4.2 ± 0.4	2.1 ± 0.2	20.2 ± 4.7	[36]
***Epichloë bromicola***	*Hordeum brevisubulatum*	0.7	5.17 ± 0.06	2.87 ± 0.17	19.5 ± 1.1	[29]
***Epichloë calamagrostidis***	*Brachypodium*	1.1–1.3	3.3–4.9	1.6–2.5	18–35	[37]
***Epichloë bromicola***	*Hordeum brevisubulatum*	0.8 ± 0.02	5.2 ± 0.1	2.9 ± 0.2	19.5 ± 1.1	[15]

nt = not tested.

**Table 2:** Colony diameters (mm) of XJE1, XJE2, and XJE3 grown at different pH values for 4 weeks.

pH Value	XJE1		XJE2		XJE3	
	Range	Mean ± SD	Range	Mean ± SD	Range	Mean ± SD
4	1.9–2.2	2.08 ± 0.13 d	2.0–2.6	2.36 ± 0.23 cd	1.5–2.0	1.82 ± 0.192 d
5	2.7–3.1	2.90 ± 0.15 bc	2.7–3.8	3.18 ± 0.53 b	2.4–2.9	2.66 ± 0.21 c
6	2.6–3.4	2.98 ± 0.32 bc	2.9–3.5	3.32 ± 0.25 b	2.3–3.0	2.74 ± 0.27 c
7	2.1–4.1	3.30 ± 0.78 b	3.6–3.8	3.72 ± 0.08 a	3.6–4.2	4.06 ± 0.26 b
8	4.4–4.8	4.56 ± 0.15 a	2.4–3.1	2.72 ± 0.26 c	4.2–5.0	4.68 ± 0.33 a
9	2.9–3.2	3.18 ± 0.31 b	2.2–2.7	2.50 ± 0.20 cd	3.5–4.6	4.28 ± 0.44 b
10	2.4–2.7	2.54 ± 0.13 cd	2.0–2.5	2.20 ± 0.19 d	2.5–3.0	2.8 ± 0.19 c

Note: Means marked with different letters (a, b, c, and d) in the same column were significantly different (*p* < 0.05).

**Table 3:** Colony diameters (mm) of XJE1, XJE2, and XJE3 grown at different NaCl concentrations for 4 weeks.

NaCl Concentration (mol/L)	XJE1		XJE2		XJE3	
	Range	Mean ± SD	Range	Mean ± SD	Range	Mean ± SD
0.1	1.9–2.7	2.34 ± 0.32 b	3.5–4.4	3.90 ± 0.32 a	3.3–4.2	3.84 ± 0.34 b
0.3	2.6–4.2	3.48 ± 0.69 a	3.2–5.0	3.84 ± 0.71 a	5.0–5.5	5.20 ± 0.19 a
0.5	1.8–2.4	1.96 ± 0.25 b	2.1–2.8	2.30 ± 0.29 b	1.2–1.5	1.34 ± 0.11 c

Note: Means marked with different letters (a, b and c) in the same column were significantly different (*p* < 0.05).

**Table 4:** Mating type and alkaloid genes in the genomes of the XJE, XJE2, and XJE3 strains isolated from *Hordeum brevisubulatum*.

Gene	Present or Absent in the Endophytic Genome			Gene	Present or Absent in the Endophytic Genome		
	XJE1	XJE2	XJE3		XJE1	XJE2	XJE3
Segments of the perA gene				Indole-diterpene (*IDT/LTM*) genes			
*perA*-A1	+	+	+	*idtG*	+	+	+
*perA*-T1	+	−	−	*idtM*	+	+	+
*perA*-C	+	+	+	*idtC*	+	+	+
*perA*-A2	+	+	+	*idtP*	−	−	−
*perA*-M	+	−	−	*idtO*	+	+	+
*perA*-T2	+	+	+	*idtF*	+	+	+
*perA*-R*	+	−	−	*idtE*	−	−	−
*perA*-△R*	−	−	−	*idtK*	+	+	+
Mating type genes				*idtB*	+	+	+
*mtBA*	+	−	−	*idtS*	+	+	+
*mtAC*	+	+	+	*idtJ*	−	−	−
Ergot alkaloid (EAS) genes				Loline (*LOL*) genes			
*dmaW*	−	−	−	*lolA*	+	−	−
*easF*	+	+	+	*lolC*	+	−	−
*easE*	+	+	+	*lolD*	−	−	−
*easC*	−	−	−	*lolE*	+	−	−
*easD*	+	+	+	*lolF*	+	−	−
*easA*	+	+	+	*lolO*	+	+	−
*easG*	+	+	+	*lolP*	−	−	−
*cloA*	+	+	+	*lolT*	−	−	−
*lpsA*	+	+	+	*lolU*	+	−	−
*lpsB*	+	+	+	*lolM*	+	−	−
*easH*	+	+	+	*lolN*	+	−	−
*lpsC*	+	+	+				
*easO*	−	−	−				
*easP*	−	−	−				

+: gene present in the endophytic genome, −: gene absent from the endophytic genome.

## Data Availability

The original contributions presented in this study are included in the article. Further inquiries can be directed to the corresponding author.
